# How to Get Along

**Published:** 1881-07

**Authors:** 


### HOW TO GET ALONG.

Never stop to tell stories in business hours.

If you have a place of business be found there when wanted.

No man can get rich sitting around stores and saloons.

Never "fool" in business matters.

Have order, system, regularity, and also promptness.

Do not meddle with business you know nothing about.

Do not kick every one in your path.

More miles can be made in a day by going steadily than stopping.

Pay as you go.

A man of honor respects his word as he does his bond.

Help others when you can, but never give when you cannot afford to, simply because it is fashionable.

Learn to say no. No necessity of snapping it out dog fashion, but say it firmly and respectfully.

Use your own brains rather than those of others.

Learn to think and act for yourself.

Advertise your business. Don't be afraid of a liberal use of printer's ink.

Respecting the Sabbath.—The people of a New Hampshire town are so fearfully lazy that, when the wife of a minister who had just settled in that town asked a prominent citizen if the inhabitants generally respected the Sabbath and refrained from business, he replied :

"Confound it, ma'am, they don't do enough work in a whole week to break the Sabbath if it was all done on that day."

